# Research on State Recognition Technology of Elevator Traction Machine Based on Modulation Feature Extraction

**DOI:** 10.3390/s22239247

**Published:** 2022-11-28

**Authors:** Dongyang Li, Jianyi Yang, Yong Liu

**Affiliations:** 1 College of Information Science and Electronic Engineering, Zhejiang University, Hangzhou 310013, China; 2 Hangzhou Special Equipment Inspection and Research Institute, Hangzhou 310051, China; 3 College of Control Science and Engineering, Zhejiang University, Hangzhou 310013, China

**Keywords:** vibration signal, traction machine, feature extraction, state identification

## Abstract

Vibration signal analysis of the traction machine is an important part of the current rotating machinery state recognition technology, and its feature extraction is the most critical step. In this study, the time-frequency characteristics of the vibration of the traction machine under different elevator running directions, running speeds and load weights are analyzed. The novel demodulation method based on time-frequency analysis and principal component analysis (DPCA) is used to extract the periodic modulated wave signal. In order to compare different influence of background noise and unknown frequency influence, the Fast Fourier Transform (FFT) and Short Time Fourier Transform (STFT) methods are used to extract the characteristics of the traction machine vibration signal, respectively. Under different load conditions, it is difficult to observe the obvious differences and similarities of the vibration signals of the traction machine by time-frequency method. However, the DPCA demodulation method provides a guarantee for the reliability and accuracy of the state identification of the traction machine.

## 1. Introduction

Elevator is a large-scale complex equipment integrating mechanical, electrical and control. If it breaks down, it will directly affect the safe and efficient operation of the elevator. Nowadays, elevators are used more and more frequently in daily life and production. Therefore, the number of elevators shows a trend of continuous growth [1], and the accompanying elevator failure and maintenance problems are becoming more and more prominent [2,3]. As the power device of the elevator, the traction machine determines whether the elevator can operate normally. With the vigorous development of science and technology, the state recognition technology of elevator traction machine is also constantly improving [4,5].

In recent years, the fault diagnosis technology of elevator traction machine with the artificial intelligence [6] or image processing [7] has been widely applied and developed. However, these methods face problems, such as non-universality of diagnostic model, high cost of model training, and requirement for massive fault samples. In addition, the selection of fault features is also of great significance to the optimization of diagnosis model.

Traction machine is a complex mechanical structure, which is closely connected by various parts. Therefore, the state identification of the traction machine can be diagnosed by various signals, such as vibration, noise, current, temperature, braking torque, speed, and power. Many useful information is hidden in the vibration signal of the traction machine [8]. These signal characteristics can reflect the working condition of the equipment. By analyzing the vibration characteristics of the equipment, the safety operation, accident prevention and maintenance cost reduction can all be accomplished.

Based on the vibration signals, a lot of research have been done in which signal feature extraction methods are the most important section of fault diagnosis [9,10]. The time domain method and frequency domain method have been commonly used in early fault diagnosis engineering [11]. Filtering, amplification, statistical feature calculation, correlation analysis, and other time-domain signal processing are referred to as time-domain signal analysis. However, it only reflects the change of amplitude with time and lack frequency bands information. The frequency domain analysis method is to describe the raw signal in the frequency domain, which is more intuitive than the time domain analysis method. However, traditional frequency domain analysis might fail to extract the characteristics information of traction machines due to the heavy background noise and complicated excitation sources [12]. Therefore, only relying on the frequency domain analysis method is far from meeting the current requirements of traction machine fault diagnosis. This brings huge challenges to the status identification and fault diagnosis of traction machines. Therefore, a time-frequency combination processing method has been proposed. Short-time Fourier transform (STFT) [13,14] and wavelet transform (WT) [15] are the common used processing tools with fine time localization and frequency resolution. These methods are realized by superposition of Fourier transform in different fixed window length. However, due to lacking self-adaptability, the quality of feature extraction might be affected by the selection of window function or wavelet basis function.

In addition, the above methods can extract the characteristics of vibration signals, but the collected vibration signals usually contain background noise and unknown frequency interference. To eliminate noise component and extract the fault feature information of raw vibration signals, several demodulation techniques have been applied to past research, such as Hilbert transform (HT) [16], empirical mode composition (EMD) [17], spectral kurtosis (SK) [18], nonstationary analysis [19,20,21], and cyclostationary analysis [22,23,24]. These methods have been applied to modulation frequency extraction already, which noted the modulation mechanism in a rotating machine.

Feng et al. [25,26] proposed an adaptive iterative generalized demodulation method to extract the modulation features in nonstationary analysis. The vibration characteristics of hydraulic turbine and planetary gearbox have been successfully found in the joint time-frequency domain. Most vibration signals of traction machine are non-stationary signals, but they are cyclostationary signals, namely, the correlation function of traction machine signals is periodic function of time. In view of the cyclostationary analysis theory, a variety of methodologies have been proposed, in which cyclic modulation spectrum (CMS) and fast spectral correlation (Fast-SC) are two typical cyclostationary tools [22]. However, they did not gain its deserved attention because of high computational cost.

Wang et al. [27] improved the cyclostationary methods with an application of Teager Kaiser energy operator (TKEO), which can enhance fault feature recognition with low computational burden. Song et al. [28,29] proposed a demodulation method based on time-frequency analysis (TSA) and principal component analysis (PCA) and applied it to the modulation frequency extraction of pump and permanent magnet synchronous motor (PMSM). Moreover, due to dimensionality reduction of time-frequency distribution matrix, the burden of high computational cost was greatly relieved. The main process of the algorithm is as follows: Firstly, the raw vibration signal is transformed into time-frequency domain by STFT. Then, the PCA method is used to reduce the dimensionality of the time-frequency spectrum in order to extract the eigenvalues of the principal components. Finally, the principal components are reconstructed to obtain the modulation signals.

Among the above demodulation methods, it could be found that the demodulation method base on PCA (DPCA) has great potential for applications in traction machine. In addition, although the fault diagnosis technology of elevator traction machine based on artificial intelligence or image processing has been widely applied and developed. However, few investigations have been done to extract and analyze the modulation features of traction. The modulation mechanism of traction machine has also rarely been involved. These above issues have greatly hindered the development of elevator fault diagnosis technology.

In this paper, the modulation characteristics of the traction machine vibration signal were extracted through a demodulation method based on time-frequency analysis and principal component analysis (DPCA). The characteristics extracted by DPCA is more prominent under the interference of background noise and unknown frequency, which is helpful to the state identification of the traction machine. The principle of signal demodulation method and experiential setting are introduced respectively in Section 2. In Section 3, the vibration signal of the traction machine is processed by FFT, STFT and DPCA methods. The influence of different working conditions on the vibration of traction machine is discussed, which shows the superiority of the demodulation technology. Finally, the conclusions are drawn in Section 4.

## 2. Methodology

### 2.1. DPCA Method

To identify the state of machinery, the mainly three steps are as follows: acquisition of monitoring signals, feature extraction of monitoring signals, and pattern recognition and diagnosis of the state are carried out. For the traction machine state recognition technology, the extraction of state features is hard work, which directly affects the accuracy of state diagnosis and the reliability of early prediction.

The state parameters of the tractor during operation are hidden in the raw signals. Therefore, the extraction of the state parameters has become an important factor affecting the accuracy of the state identification. Based on the feature extraction, various signal processing techniques have been developed, which mainly involved time domain analysis, frequency domain analysis, time-frequency analysis, etc. [30]. Although the above methods can extract the features of vibration signals, the collected vibration signals usually contain background noise and unknown frequency interference. The amplitude demodulation process (also known as high frequency resonance, resonance demodulation or envelope analysis) separates low frequency from high frequency background noise [31]. In this paper, the DPCA method was adopted for feature extraction [28]. DPCA algorithm mainly includes: time-frequency analysis, principal component analysis and feature extraction.

(1) Time frequency analysis.

When the traction machine operates stably, its key modulation component is modulation signal. The single component modulation signal of the traction machine can be expressed in Equation (1), which is mainly composed of modulation signal and carrier signal.

(1) $$x(t)=x_{m}(t)x_{c}(t)$$

where *x*(*t*) is the amplitude modulation signal of the traction machine, *x_m_*(*t*) is the modulation signal, and *x_c_*(*t*) is the carrier signal.

The time-frequency distribution of the monitoring signal can be expressed in Equation (2).

(2) $$P_{X}(f,t)=\int_{-\infty }^{\infty }x_{m}(\tau )x_{c}(\tau )w(t-\tau )e^{-j2\pi f\tau }d\tau$$

where *P_X_* (*f, t*) is the time-frequency distribution function of the monitoring signal, and *w* (*t*) is the window function of the STFT.

The STFT of the modulation signal model of the traction machine can be approximated as follows, as shown in Equation (3).

(3) $$\int_{-\infty }^{\infty }x_{m}(\tau )x_{c}w(t-\tau )e^{-j2\pi f\tau }d\tau \approx x_{m}(\tau )\int_{-\infty }^{\infty }x_{c}(\tau )w(t-\tau )e^{-j2\pi f\tau }d\tau$$

The time spectrum of the modulated signal is further simplified to obtain Equation (4).

(4) $$P(f,t)\approx x_{m}(t)\int_{-\infty }^{\infty }x_{c}(\tau )w(t-\tau )e^{-j2\pi f\tau }d\tau =x_{m}(t)P_{C}(f,t)$$

where *P* (*f, t*) represents the time-frequency distribution function of the detection signal, *P_C_* (*f, t*) represents the time-frequency distribution function of the carrier signal.

(2) Principal component analysis.

Principal component analysis is a classical data dimensionality reduction method, which is mainly realized by the following algorithms.

Firstly, the covariance matrix is solved. The matrix formula is shown in Equation (5).

(5) $$P_{\mathrm{cov}}=\mathrm{cov}(P(t,f))$$

where *P*_cov_ represents the covariance matrix of the time-frequency distribution matrix, cov() represents the covariance operator.

Secondly, there is eigenvalue decomposition. As shown in Equation (6).

(6) $$\left[V,U\right]=\mathrm{eig}(P_{\mathrm{cov}})$$

where eig() represents the eigenvalue decomposition operator. **V**, **U** represent eigenvalue matrix and eigenvector matrix respectively.

Thirdly, eigenvalue selection. The order of the selected eigenvalue is determined by the maximum value of the difference spectrum, as shown in Equation (7).

(7) $$k\geq i{|}_{\max(\delta_{i}=(\lambda_{i}-\lambda_{i+1}))}$$

where *k* represents the order of the selected eigenvalue, δ*_i_* represents the difference spectrum value.

Finally, principal component reconstruction. The corresponding principal component modulation signal PPC*_i_*(*t*) can be obtained, as shown in Equation (8).

(8) $${\mathrm{PPC}}_{i}(t)=P(t,f)u_{i}$$

(3) Feature extraction.

The principal component analysis method can be used to obtain the principal component of the monitoring signal, which includes the low-frequency modulation component of the monitoring signal. The characteristic modulation frequency can be extracted by frequency analysis, as shown in Equation (9).

(9) $$P_{i}(f)=\int_{-\infty }^{\infty }{\mathrm{PPC}}_{i}(t)e^{-j2\pi ft}dt$$

### 2.2. Elevator Traction Machine Parameters

The model of tractor selected in the experiment is GETM3.DM. The detailed parameters are listed in Table 1.

### 2.3. Equipment Selection

**Figure 1:**
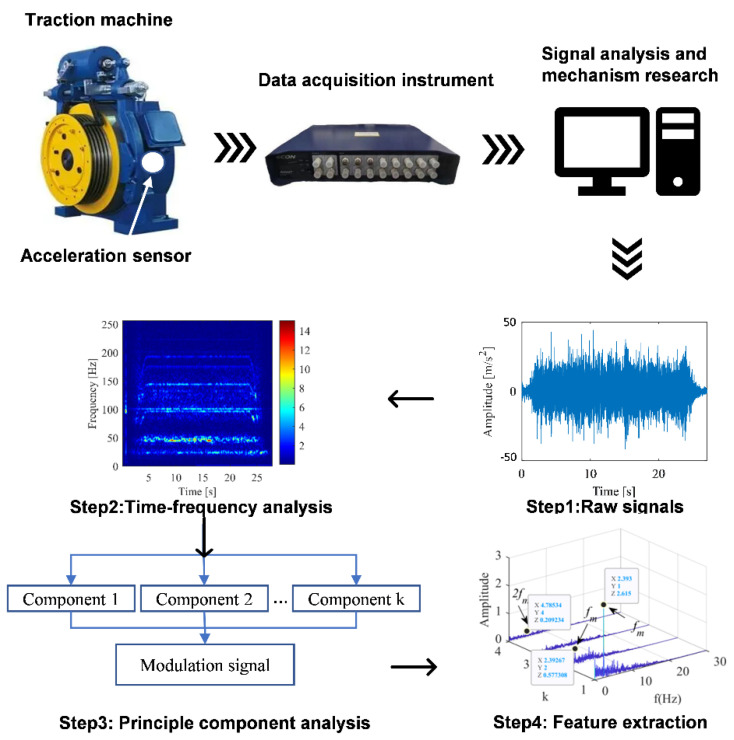
Flow chart of traction machine vibration signal acquisition and analysis.

The test system was used for the state identification research of elevator traction machine, as shown in Figure 1. The instruments included vibration acceleration sensor, data acquisition instrument, computer, and other auxiliary instruments. The acceleration sensor was fixed to the traction machine, and the vibration signals collected by the sensor were transmitted to the data acquisition instrument.

### 2.4. Test Conditions

In order to realize the recognition of different states of the traction machine, the test conditions involved in this paper are listed in Table 2.

## 3. Case Analysis

### 3.1. Analysis of Influence of Elevator Running Speed on Main Engine Vibration

**Figure 2:**
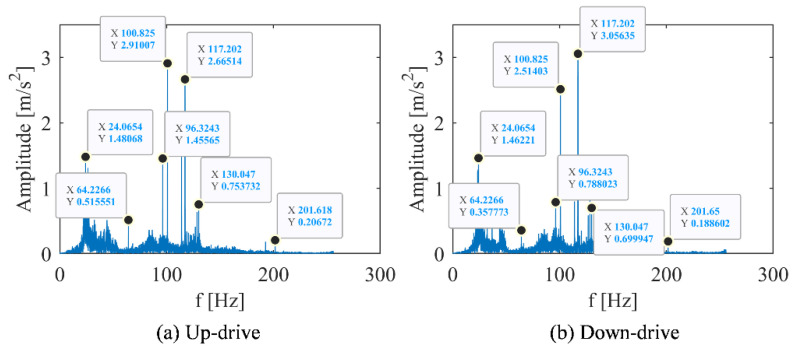
Vibration spectrum diagram of main engine at elevator running speed of 1 m/s. (**a**) Motor with up-drive condition. (**b**) Motor with down-drive condition.

**Figure 3:**
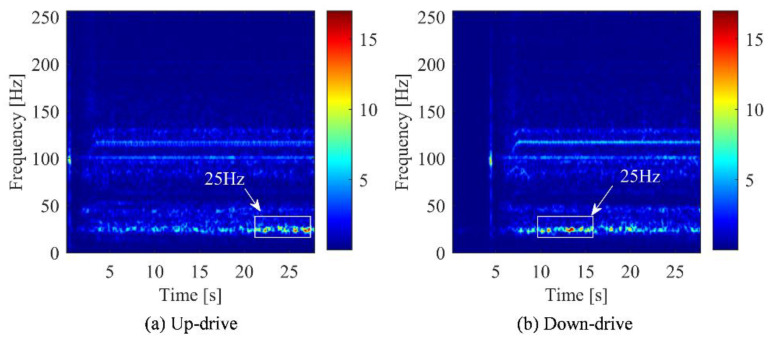
Time-frequency diagram of main engine vibration at elevator running speed of 1 m/s. (**a**) Motor with up-drive condition. (**b**) Motor with down-drive condition.

**Figure 4:**
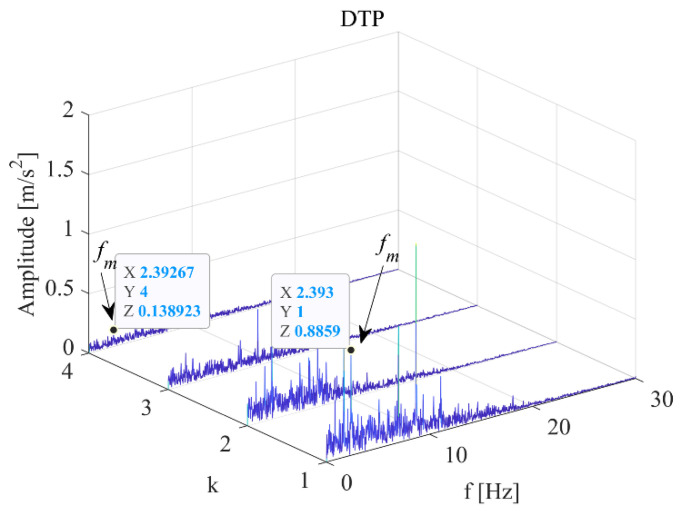
Vibration demodulation diagram of main engine when elevator speed is 1 m/s.

**Figure 5:**
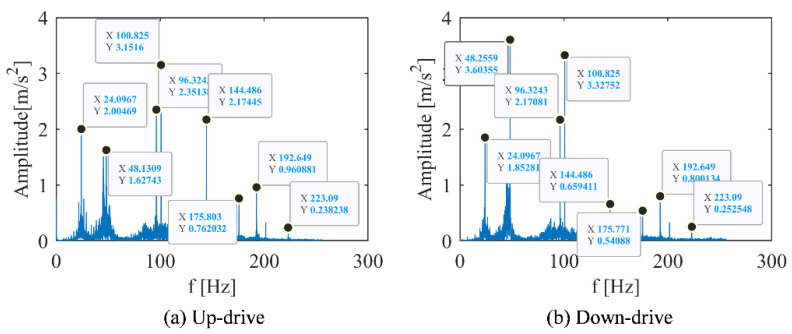
Vibration spectrum of main engine in different running directions of elevator (2.4 m/s). (**a**) Motor with up-drive condition. (**b**) Motor with down-drive condition.

**Figure 6:**
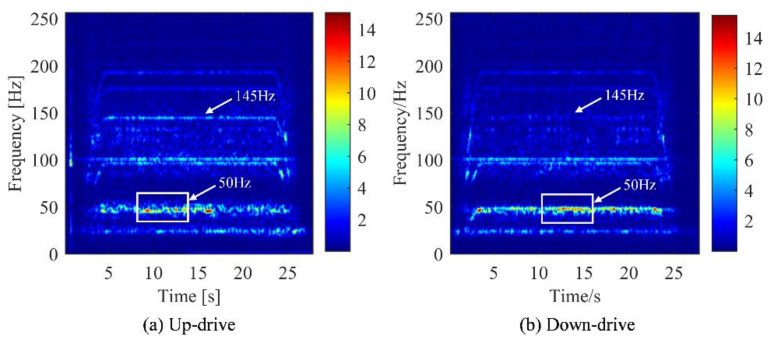
Time frequency diagram of main machine vibration in different operating directions of elevator (2.4 m/s). (**a**) Motor with up-drive condition. (**b**) Motor with down-drive condition.

**Figure 7:**
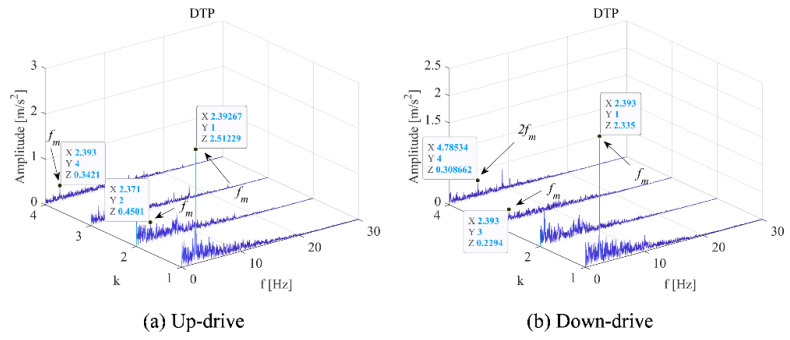
Vibration demodulation diagram under different operating conditions (2.4 m/s). (**a**) Motor with up-drive condition. (**b**) Motor with down-drive condition.

To compare features extracted by the different signal analysis methods, FFT was used to transform the time domain signal into spectrum domain. Their peaks value of the spectrum under different conditions are recorded in Table 3 and Table 4. Under the working condition of 1 m/s, the frequency spectrum, time-frequency spectrum, and DPCA result are shown in Figure 2, Figure 3 and Figure 4, respectively. Under the working condition of 2.4 m/s, the frequency spectrum, time-frequency spectrum, and DPCA result are shown in Figure 5, Figure 6 and Figure 7, respectively.

With the comparation of the FFT results, it can be found that operating speed has an impact on the amplitude of the vibration spectrums. The amplitude of each frequency under low-speed operation (1.0 m/s) was lower than the amplitude of each frequency under normal operation (2.4 m/s), as shown in Figure 2 and Figure 5. In general, the peak frequency in vibration spectrum will increase with acceleration of the traction machine.

In the time-frequency spectrum shown in Figure 3, it can be found that the prominent frequency of the elevator machine at the operating speed of 1 m/s is approximately 25 Hz. While the prominent vibration frequency under normal speed (2.4 m/s) is approximately 50 Hz, as shown in Figure 6. This characteristic is positively related to the operating speed of the traction machine, which can be regard as the main feature of elevator vibration. The vibration level of the traction machine can be evaluated by the amplitude change of this frequency band in the time-frequency spectrum.

Through comparative analysis, the DCPA results shown in Figure 4 and Figure 7a. The k refers to the serial number of principle frequency bands selected by Equation (7). The modulated frequency of elevator traction machine is indicated by *f*_m_, which can be found in each modulation spectrum. It can be observed that when *k* = 1, the amplitude modulation difference of *f*_m_ under two working conditions is 2.58 times. This value is approximate to the speed ratio under two working conditions. Therefore, the mechanism of vibration level-up caused by the operating speed-up is the increase of modulation effect in principle frequency bands.

As a result, for the working conditions with obvious differences, such as the influence of different operating speeds of the elevator on the vibration signal of the traction machine, the difference between the two states could be obtained by analyzing the frequency-domain diagram through the FFT. The time-frequency diagram and the demodulation diagram can more clearly highlight the difference and complete the identification of the state of the traction machine.

### 3.2. Analysis on the Influence of Elevator Running Direction on the Vibration of Main Engine

The spectrum analysis about different running direction is shown in Figure 5 and Table 4. It can be found that the vibration response was largest at 100.8 Hz under up-drive operation, while the largest vibration response under down-drive operation was at 48.1 Hz.

When the frequency was 48.1 Hz, the peak value of the downlink is much larger than that of the uplink, which was twice that of the downlink. As the frequency was 144.5 Hz, the peak value of the uplink was much larger than that of the downlink, which was three times that of the downlink. Except for 48.1 Hz and 144.5 Hz, the characteristic frequency of the most obvious peak under the two working conditions of the elevator was basically unchanged, and the height of the main peak slightly changed.

According to the analysis of Figure 6, the frequency (rotation speed) of the elevator gradually increased from the start to a certain state and then remains stable. After a cycle of operation, the frequency gradually decreased. Comparing (a) and (b) in Figure 6, it can be found that the peak value of the uplink was greater than that of the downlink at 145 Hz, while the difference in other frequency bands were not significant.

From the time-frequency spectrum shown in Figure 6, it can only be concluded that the difference between the two working conditions was the most significant at the frequency of 145 Hz. However, it was not enough to support the identification of the elevator’s up and down conditions. Therefore, based on the spectrum analysis of STFT, the modulation signal in the vibration signal of the traction machine was extracted by the PCA technology.

The vibration demodulation diagram of the main engine under different operating conditions of the elevator in Figure 7 was analyzed. When *k* = 1, there was little difference between the up-working condition and the down-working condition; when *k* = 2, the frequency modulation of one *f*_m_ is generated more in the upstream working condition than in the downstream working condition; when *k* = 3, a *f*_m_ frequency modulation is generated in the downstream working condition more than in the upstream working condition; and when *k* = 4, the uplink has a frequency modulation of *f*_m_ and the downlink has a frequency modulation of 2*f*_m_.

For the up and down working conditions of the elevator, the influence on the traction machine was not obvious. Only the frequency domain diagram and the time-frequency diagram cannot accurately distinguish the two working conditions. Therefore, the demodulation method was used to separate the signal from the raw signal, highlight the weak state characteristic signal, and distinguish the states of different working conditions.

### 3.3. Analysis of Influence of Elevator Load on Main Engine Vibration

**Figure 8:**
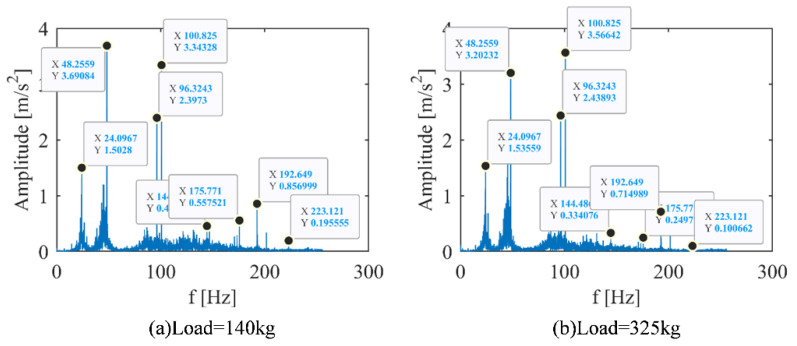
Vibration spectrum diagram of elevator main engine with different loads. (**a**) Motor running under 140 kg load condition. (**b**) Motor running under 325 kg load condition.

Taking the behavior under the elevator as an example, the measured time domain diagram was transformed into a spectrum diagram by FFT, as shown in Figure 8.

**Figure 9:**
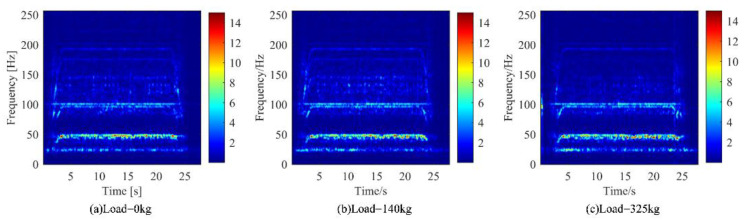
Vibration time-frequency diagram of down main machine under different loads of elevator. (**a**) Motor running under no-load condition. (**b**) Motor running under 140 kg load condition. (**c**) Motor running under 325 kg load condition.

From the Figure 5b and Figure 8 and Table 4 and Table 5, it was hard to identify different working conditions only by using the frequency domain diagram. Then, the time-frequency spectrums obtained by STFT was analyzed, as shown in Figure 9.

However, it was also difficult to distinguish the working conditions of different loads by time-frequency spectrums. Therefore, based on the spectrum obtained by STFT, the modulation signal in the vibration signal of the traction machine was extracted by the DPCA method.

**Figure 10:**
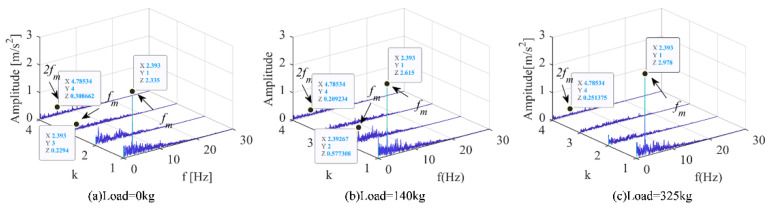
Vibration demodulation diagram of down main machine under different loads of elevator. (**a**) Motor running under no-load condition. (**b**) Motor running under 140 kg load condition. (**c**) Motor running under 325 kg load condition.

From the vibration demodulation diagram of the elevator main engine in Figure 10, it can be found that the frequency modulation of 2*f*_m_ was less than that of the other two working conditions when *k* = 1, and the frequency modulation of 2*f*_m_ was less than that of the other two directions when *k* = 2. When the load was 325 kg and *k* = 3, the frequency modulation of *f*_m_ was less than that of other working conditions. In addition, the amplitude corresponding to the common frequency modulation in the three working conditions increases with the increase of the load.

According to the working conditions of different loads of the elevator, the influence on the traction machine is not obvious. It was difficult to distinguish the two working conditions only through the frequency-domain diagram and the time-frequency diagram. Therefore, the demodulation method is used to separate the signal from the raw signal, highlight the weak state characteristic signal, and distinguish the states of different working conditions.

## 4. Conclusions

In this paper, the application of the DCPA method provides an alternative way to realize fast and effective condition monitoring of a traction machine, which could be extended to detect other background interference and typical faults. The conclusion is as follows:

(1) For the influence of different operating speeds of the elevator on the vibration signal, the difference can be obtained by analyzing the frequency-domain diagram through the FFT. The time-frequency diagram and the demodulation diagram can more clearly highlight the difference and complete the identification of the state of the traction machine. The amplitude modulation ratio of *f*_m_ is approximate to the speed ratio under working conditions for different speeds;
(2) For the up and down working conditions of the elevator, the frequency domain diagram and the time-frequency diagram cannot accurately distinguish the two working conditions. The DPCA demodulation method could highlight the weak state characteristic signal and distinguish the states of different working conditions;
(3) Under different load conditions, it is difficult to observe the obvious differences and similarities of the vibration signals of the traction machine by time-frequency method. However, the DPCA demodulation method can effectively solve the influence of background noise and unknown frequency interference of the traction machine vibration signal. With the increase of load, the amplitude modulation of shaft frequency (*f*_m_) increases;
(4) The state identification technology discussed in this paper involved a healthy traction machine under various operation. The state identification of traction machines with different faults will be carried out in future work.

## Figures and Tables

**Table 1:** Parameters of elevator traction machine.

Parameter	Value
Model	GETM3.DM
Moment of inertia [kg·m^2^]	4.4
Pulley diameter [mm]	400
Rated voltage [V]	513
Rated current [A]	12.6
Rated power [kW]	9.7
Rated speed [rpm]	168
Rated frequency [Hz]	28

**Table 2:** Test conditions.

Working Condition	Classification		
Different running directions	(a) Elevator up	(b) Elevator down	
Different operating speeds	(a) 1 m/s	(b) 2.4 m/s	
Different loads	(a) no-load	(b) 140 kg	(c) 325 kg

**Table 3:** Frequency domain peak value of vibration response at running speed of 1 m/s.

Peak Sequence Number	1	2	3	4	5	6	7
Frequency (Hz)	24.1	64.2	96.3	100.8	117.2	130.0	201.6
Up-drive (m/s^2^)	1.48	0.51	0.52	1.46	2.91	2.67	0.64
Down-drive (m/s^2^)	1.46	0.49	0.36	0.79	2.51	3.06	0.82

**Table 4:** Frequency domain peak value of vibration response in different running directions of elevator.

Peak Sequence Number	1	2	3	4	5	6	7	8
Frequency (Hz)	24.1	48.1	96.3	100.8	144.5	175.8	192.6	223.1
Up-drive (m/s^2^)	2.00	1.63	2.35	3.15	2.17	0.76	0.96	0.24
Down-drive (m/s^2^)	1.85	3.60	2.17	3.33	0.66	0.53	0.80	0.25

**Table 5:** Frequency domain peak value of vibration response of elevator with different loads.

Peak Sequence Number	1	2	3	4	5	6	7	8
Frequency (Hz)	24.1	48.1	96.3	100.8	144.5	175.8	192.6	223.1
0 kg	1.85	3.60	2.17	3.33	0.66	0.53	0.80	0.25
140 kg	1.50	3.69	2.40	3.34	0.42	0.56	0.86	0.20
325 kg	1.54	3.20	2.44	3.57	0.33	0.25	0.71	0.28

## Data Availability

Data will be made available on request.
